# Population abundance in arctic grayling using genetics and close‐kin mark‐recapture

**DOI:** 10.1002/ece3.7378

**Published:** 2021-04-02

**Authors:** Samuel Prystupa, Gregory R. McCracken, Robert Perry, Daniel E. Ruzzante

**Affiliations:** ^1^ Department of Biology Dalhousie University Halifax NS Canada; ^2^ Fish and Wildlife Division Department of Environment Whitehorse, Yukon Canada; ^3^ Present address: Department of Zoology University of Otago Dunedin New Zealand

**Keywords:** census size, CKMR, parent offspring pairs, population abundance, population structure, Thymallus arcticus

## Abstract

Arctic Grayling (*Thymallus arcticus*) are among the most widely distributed and abundant freshwater fish in the Yukon Territory of Canada, yet little information exists regarding their broad and fine‐scale population structures or the number and size of these populations. The estimation of population abundance is fundamental for robust management and conservation, yet estimating abundance in the wild is often difficult. Here, we estimated abundance of an Arctic Grayling population using multiple genetic markers and the close‐kin mark‐recapture (CKMR) method. A total of *N* = 1,104 Arctic Grayling collected from two systems in Yukon were genotyped at 38 sequenced microsatellites. We first identified structure and assessed genetic diversity (effective population size, ${\hat{N}}_{e}$). Collections from one of the systems (Lubbock River) comprised adults and young‐of‐the‐year sampled independently allowing the identification of parent–offspring pairs (POPs), and thus, the estimation of abundance using CKMR. We used COLONY and CKMRsim to identify POPs and both provided similar results leading to indistinguishable estimates (95% CI) of census size, that is, ${\hat{N}}_{c(COLONY)}$ = 1858 (1259–2457) and ${\hat{N}}_{c(CKMRsim)}\;=\;$1812 (1229–2389). The accuracy of the population abundance estimates can in the future be improved with temporal sampling and more precise age or size‐specific fecundity estimates for Arctic Grayling. Our study demonstrates that the method can be used to inform management and conservation policy for Arctic Grayling and likely also for other fish species for which the assumption of random and independent sampling of adults and offspring can be assured.

## Graphical abstract

We estimate population abundance of Arctic Grayling using the close‐Kin mark‐recapture method and a newly developed suite of 38 sequenced species specific microsatellite DNA markers. This is done after assessing population structure and estimating effective sizes. We suggest the approach including the added night gained by estimating the ration *N_e_*/*N_c_* can be added to the conservation and management toolkit for this and potentially other freshwater species where random sampling and independence of sampling of adults and potential offspring can be assured.

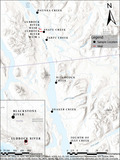

## 1 INTRODUCTION

Arctic Grayling (*Thymallus arcticus*) are among the most widely distributed and abundant freshwater fish in Canada's Yukon Territory (Department of Fisheries and Oceans (DFO) 2015). The species is important for subsistence fishing by First Nations and is also considered important for recreational fishing (Northcote, 1995). The cumulative effects of increasing anthropogenic effects associated with climate change, and land development have placed the species under increasing stress in the region, bringing the need to improve our understanding of its life history and ecology into sharp focus. Yet, despite the urgent need to better understand their life history for management and conservation purposes, grayling remain one of the least studied species in the territory (Jessup & Millar, 2012) and little information exists regarding their broad and fine‐scale population structures. The paucity of research can, in part, be attributed to the inherent difficulties in studying an animal with a complex life history that involves large scale seasonal movements from summer feeding and staging areas in large rivers and lakes to late winter and spring spawning sites in smaller tributaries where they form spawning aggregations and become vulnerable to over harvesting (Jessup & Millar, 2012). In Yukon, Arctic Grayling populations appear to have declined over the last several decades leading to the enforcement of fishing restrictions, including maximum size limit, mandatory use of barbless hooks, and reduced catch and possession limits in 2001 (Environment Yukon, 2010; Foos et al., 2014). Yet, despite the importance of the fishery for the region, detailed information on population status (e.g., abundance, trends, genetic diversity) necessary for the implementation of robust management and conservation policy is generally lacking or deficient (Jessup & Millar, 2012).

Genetically distinct populations of grayling are known to intermix during their summer and winter staging periods making individual populations difficult to quantify during this time. Yet in the spring adult grayling exhibit natal philopatry, concentrating their numbers as they migrate large distances, away from their feeding and staging areas, to return to their natal spawning beds (Armstrong, 1986; Lashmar & Ptolemy, 2002; McPhail & Lindsey, 1970). To overcome census difficulties, Yukon fisheries managers have primarily resorted to quantifying grayling during their spawning period when they will briefly separate into discrete groups (Lashmar & Ptolemy, 2002; Reilly, 2014). Such assessments are normally conducted using mark‐recapture surveys, weir counts, or snorkel surveys (Jessup, 2014; Read & Roberge, 1989). However, all of these methods are labor intensive and often insufficient for the estimation of overall abundance (Pinnix et al., 2016). They also have limited application in Yukon since typically they can only be applied in small streams with slow‐moving water and low turbidity, yet many of Yukon's streams and rivers are large, fast moving, and lack clarity. Therefore, a new approach for the estimation of population abundance is required to overcome the limitations of existing methods.

Here, we estimated Arctic Grayling population census (*N_c_*) and effective (*N_e_*) sizes and their ratio using genetic information. The approach we used to estimate census size with genetic data, known as close‐kin mark‐recapture (CKMR), is based on the principle that an individual's genotype can be considered a “recapture” of the genotypes of each of its parents (Skaug, 2001). Assuming the sampling of offspring and parents is independent of each other, the number of parent–offspring pairs genetically identified in a large collection of both groups can be used to estimate abundance when interpreted in a mark‐recapture framework (Bravington et al., 2016). Close‐kin mark‐recapture has recently been applied to estimate population abundance in Southern Bluefin Tuna (Bravington et al., 2016) and white sharks (Hillary et al., 2018) as well as *N_e_/N_c_* ratios in Southern Bluefin Tuna (Waples et al., 2018) and has recently been validated by comparing CKMR‐based estimates with census estimates based on standard mark‐recapture in Brook Trout small populations (Ruzzante et al., 2019). Major assumptions of the method include (a) that individuals are thoroughly mixed throughout the area of interest, (b) that potential parents and offspring are sampled independently of each other and that the sampling is random (Bravington et al., 2016; Ruzzante et al., 2019), though recent work showed the method to be robust to some departures from spatially uniform sampling probabilities and dispersal limitations (Conn et al., 2020). Ultimately, however, obtaining an unbiased estimate of census population size depends on the probability of capture and hence on the precise identification of the geographic scale at which populations are structured.

Arctic Grayling are well suited for the application of the close‐kin mark‐recapture for the estimation of population abundance. River populations of grayling use tributary stream for spawning, where environmental conditions for spawning and rearing are favorable (Armstrong, 1986; McPhail & Lindsey, 1970). Arctic Grayling return to the same summer habitat and may also home to their natal streams to spawn (Tack, 1980; Northcote, 1995; Lashimar & Ptolemy, 2002) with recent work reporting significant genetic divergences between and within three major river basins, and strong isolation by distance patterns, consistent with homing to natal streams in the species (Reilly, 2014). Additionally, Arctic Grayling adults will be resident on the spawning beds during the entire spawning period and their eggs hatch two to three weeks following the spawning event. Thus, both parent and offspring are readily available for sampling to conduct CKMR.

Here, we used a set of 38 newly developed sequenced microsatellite DNA markers first, to identify population structure in Arctic Grayling inhabiting two river systems in Yukon, the Lubbock and the Blackstone River systems. The Lubbock River system was chosen because of its regional importance for recreational fishing, prior knowledge on the location of Arctic Grayling spawning aggregations and its relative ease of access by road. The Blackstone River is located within the Tombstone Territorial Park in northwestern Yukon. This park has seen a dramatic increase in the number of visitors following the 1979 opening of the Dempster Highway to the town of Inuvik located north of the Arctic Circle. This has likely resulted in an increase in the stress imposed on local Arctic Grayling populations. No information currently exists, however, either on the number and size of these population(s) or on their critical spawning habitat. For the Lubbock River population, where sampling involved collection of adults and YOY, we estimated census population size (*N_c_*) using the CKMR approach. We discuss population status and its importance in informing robust management and conservation policy for Arctic Grayling.

## 2 MATERIAL AND METHODS

### 2.1 Study sites and field sampling

**FIGURE 1:**
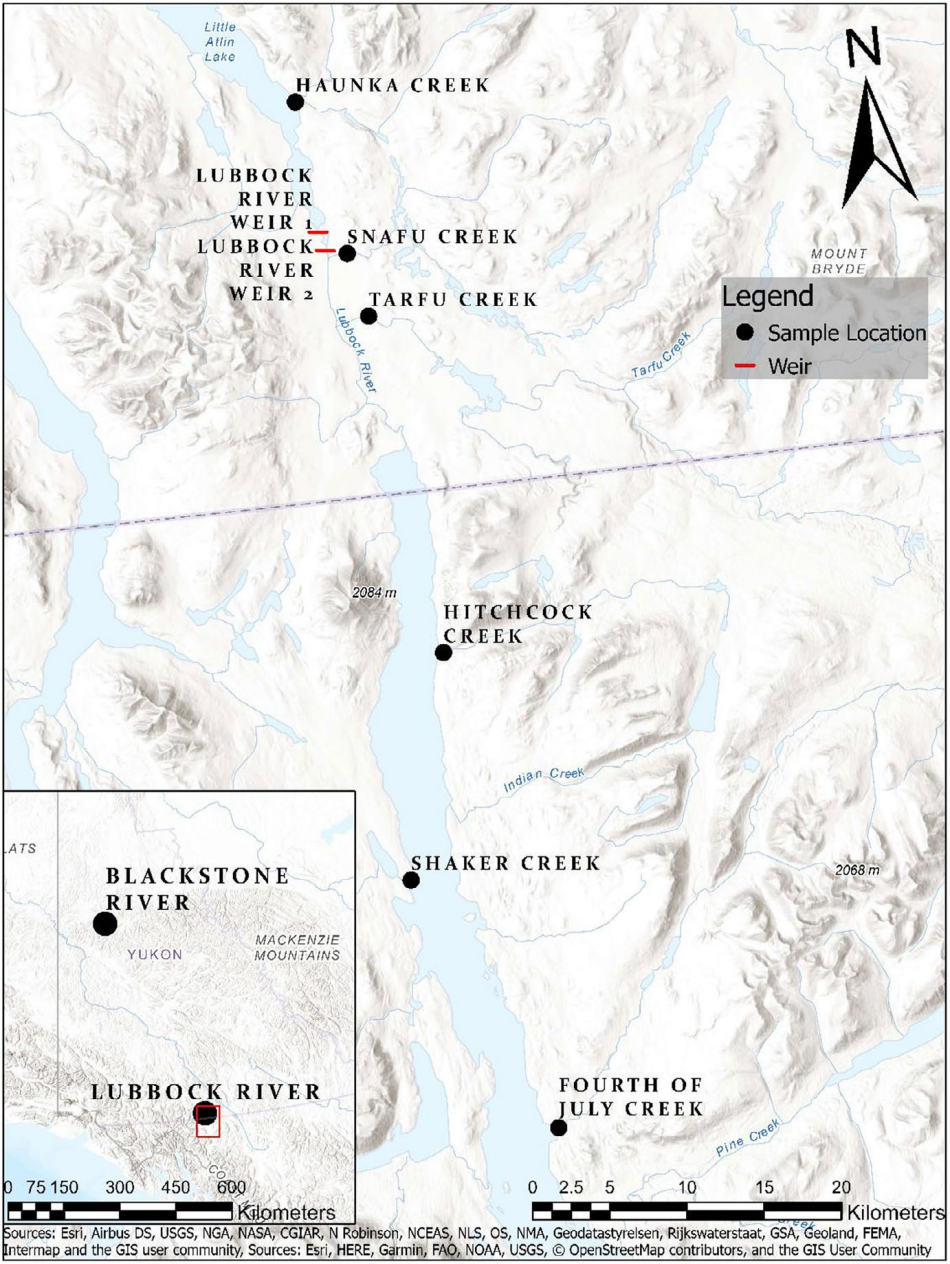
Location of the Blackstone and Lubbock systems in the Yukon Territory, Canada, sampling locations in the Lubbock system include as follows: Haunka Creek (Little Atlin Lake), Lubbock River, Snafu Creek, Tarfu Creek, and three locations in Atlin Lake: Hitchcock Creek, Shaker Creek, and Fourth of July Creek

We collected *N* = 1,104 Arctic Grayling (*Thymallus arcticus*) from two river systems in the Yukon Territory of Canada, the Lubbock River system (Pacific Ocean drainage) along the British Columbia and Yukon border and the Blackstone system (Arctic Ocean drainage) in northern central Yukon (Figure 1). In the Lubbock system, fish were collected from 7 sites separated by a maximum waterway distance of ~68 km (Table 1, Figure 1). Within this system, two methods of fish collection were used as follows: First, two weirs were set ~1 km apart in a section of the Lubbock River where fish congregate for spawning. Both weirs had gates collecting fish swimming upstream and downstream thus allowing the (nonlethal) sampling of all adult fish entering or leaving the enclosed ~1 km river section where grayling are known to congregate for spawning. Adult fish were sampled at the weirs over 10‐week period between late April and early July 2018. During that time, we also collected YOY by dip netting within this enclosed area and in the other six sites in the Lubbock system. For comparison, we also collected Young‐of‐the‐Year (YOY) from the Blackstone system in the north; these fish were collected over a 10‐day period in July 2018 by dip netting from a single site spread over ~1 km waterway distance. We measured fork length and took a fin clip sample for DNA analysis from all adults sampled at the weirs. Fin clips or the entire individual (YOY) were stored in 95% ETOH. Field collections were conducted under permit from the Yukon Department of Environment and Dalhousie University laboratory Animal Ethics protocol (I18‐16).

**TABLE 1:** Sampling locations with latitude and longitude, sample size, median fork length of individuals per location, observed (*H_o_*) and expected (*H_e_*) heterozygosity averaged across 38 microsatellite loci for each location with standard deviation, and p‐value for comparing means of observed and expected heterozygosity

River system	Sampling location	Lat. (*N*)	Long. (W)	*n*	Median Length (mm) Offspring (Adults)	*H_o_* (*SD*)	*H_e_* (*SD*)	*p*‐Value
Blackstone	Blackstone River	64.85	138.32	137	16	0.58 (0.21)	0.58 (0.216)	.975
Lubbock	Haunka Creek	60.23	133.90	69	30	0.53 (0.22)	0.47 (0.159)	.241
	Lubbock River	60.15	133.88	594	24 (342)	0.50 (0.21)	0.49 (0.197)	.789
	Snafu Creek	60.14	133.85	56	28	0.51 (0.23)	0.50 (0.194)	.766
	Tarfu Creek	60.11	133.84	70	26	0.51 (0.19)	0.49 (0.180)	.673
	Hitchcock Creek	59.91	133.80	49	23	0.44 (0.19)	0.45 (0.185)	.880
	Shaker Creek	59.78	133.86	57	31	0.50 (0.23)	0.48 (0.205)	.674
	Fourth of July Creek	59.62	133.73	72	23	0.50 (0.20)	0.49 (0.191)	.938

### 2.2 Molecular protocol

DNA analysis was performed on all *N* = 1,104 individuals (507 adults and 460 YOY from the Lubbock system, and 137 YOY from the Blackstone system). Tissue samples (~2mm fragments) were placed in a 96 well plate for tissue digestion with proteinase K (Bio Basic, Markham, ON, Canada) at a ~1:70 ratio with digestion. Digestion proceeded for ~8 hr on an incubator at 50°C (shaken at 200RPMs).

DNA was extracted using a glass milk protocol modified from Elphinstone et al., (2003) on a Perkin Elmer Multiprobe II Plus Liquid Handling System (Perkin Elmer, Waltham, MA, USA). Samples were then placed in a ~50°C oven for ~3 hr to eliminate ethanol residue. Low TE was added to remove DNA from glass milk and suspend it in solution. A subset of samples was run on 1% agarose gel stained with GelGreen (BioTium Fremont, CA, USA) to test for DNA quality and quantity.

Forward and reverse primers were designed and tested for 96 microsatellite loci (Details for microsatellite development in Appendix) from Integrated DNA Technologies (IDT, Coralville, IA, USA) tailed with Illumina (San Diego, CA, USA) sequencing primers. After testing and genotyping, 38 loci were kept for further analysis (Table S1). Sequencing was conducted in house using an Illumina MiSeq Benchtop Sequencer (Illumina, San Diego, CA, USA).

Postsequencing, individuals were demultiplexed automatically by means of index combination using the MiSeq Sequence Analysis software. Genotypes were scored with MEGASAT (Zhan et al., 2017) and subsequently verified manually. Microchecker (v2.2.3) (van Oosterhout et al., 2004) was used to identify potential null alleles and large‐allele drop‐out. GenAlEx 6.5 (Peakall & Smouse, 2012) was used to calculate percent missing data per locus and number of missing loci per individual.

### 2.3 Observed versus expected heterozygosity

Observed (*H_o_*) and expected (*H_e_*) heterozygosities were estimated for each subpopulation using Arlequin v3.0 (Excoffier & Lischer, 2010). We report mean observed and expected heterozygosity over loci. HWE was tested at *α* = 0.05 using a Welch's *t* test, to compare two means of unequal variance.

### 2.4 Population structure

The estimation of population abundance using close‐kin mark‐recapture relies on the correct identification of population boundaries. We, therefore, estimated population structure. This was done hierarchically with the software STRUCTURE v2.3.4 (Pritchard et al., 2000). We first examined the entire data set including collections from both the Blackstone and Lubbock River systems. Identified clusters were then separately subject to further analysis. STRUCTURE was run using 100,000 burn‐in steps followed by 400,000 permutations and each K value (number of genetic groups) replicated 5 times. The Evanno method (Evanno et al., 2005) as implemented in STRUCTURE HARVESTER v.0.6.92 (Earl & VonHoldt, 2012) was used to determine the most likely number of clusters at each step. The five replicates were combined into a single output using CLUMP 1.1.2 with 1,000,000 random input orders (Jakobsson & Rosenberg, 2007). The output was then visualized with DISTRUCT 1.1 (Rosenberg, 2004).

### 2.5 Effective population size and effective number of breeders

The effective number of breeders (${\hat{N}}_{b}$) was estimated for each subpopulation using LDNe (Waples & Do, 2010). A cut‐off *p*‐value of .02 was used for lowest allele frequency considered to maximize precision and reduce bias. Estimates of ${\hat{N}}_{b}$ were converted to ${\hat{N}}_{b\left(\mathrm{adj}2\right)}$ using Equation 1, and to ${\hat{N}}_{e(\mathrm{adj}2)}$ using equation 2 in Waples et al. (2014) as described here as follows:

(1) $${\hat{N}}_{b\left(\mathrm{adj}2\right)}=\;\frac{raw\;{\hat{N}}_{b}}{1.103‐0.245\;x\log\left(\frac{\mathrm{AL}}{\propto }\right)}$$

(2) $${\hat{N}}_{e(\mathrm{adj}2)}=\;\frac{{\hat{N}}_{b(adj2)}}{0.485+0.758\;x\;\log\left(\frac{\mathrm{AL}}{\propto }\right)}$$

Where AL is adult life span or number of reproductive cycles and α is age at first reproduction. Information on Arctic Grayling adult lifespan or number of reproductive cycles (AL) and age at first reproduction (∝) were taken from Clark (1992).

### 2.6 Census population size estimate by close‐kin mark‐recapture (CKMR)

Lastly, we estimated the size of the population breeding in the Lubbock River using the close‐kin mark‐recapture (CKMR) approach. This analysis was conducted with the adult individuals entering or leaving the 1 km stretch of the Lubbock River enclosed by the two weirs and the juveniles collected by dip netting within that same stretch of river and those sampled in two tributaries downstream, all of which were found to belong to the same genetic pool (See RESULTS). All individuals were sampled within a 10‐week period in the spring and summer (late April to early July) of 2018. Only individuals with ≤ 7 missing loci were considered in the parentage analysis. Among the adults, only those assumed to be mature at the time of collection were included, with the size at maturity for Arctic Grayling in the region assumed to be 275 mm (Clark, 1992).

Population abundance was, therefore, estimated for 2018 as follows:

(3) $${\hat{N}}_{c}\;\left(\mathrm{CKMR}\right)=\;\frac{2*\;N_{\mathrm{Mature}}{*N}_{\mathrm{Juvenile}}}{\left(H+1\right)}$$

Where *N*_Mature_ is the number of mature individuals genotyped in the population, *N*_Juvenile_ is the number of offspring genotyped in the population, and *H* + 1 is the number of parent–offspring pairs identified plus one for small sample bias correction. In the numerator, the 2 reflects the fact that each offspring sampled has two potential parents. In this study, all individuals (adults and offspring) were collected within a single spawning season and shortly thereafter. When this is not the case, and potential parents are collected over a protracted period spanning several years, the number of parents has to be weighed by their age‐specific relative fecundities at the time of fertilization. If collected before the spawning or fertilization event, potential parents need to be weighed also by the age‐specific survivorship rates (See Equations (2), (3) in Ruzzante et al., 2019; Waples & Feutry, 2021).

### 2.7 Parent–offspring pair estimation

We used COLONY v.2.0.6.4 (Jones & Wang, 2010) and CKMRsim (Anderson, 2020) package implemented in R (R Core Team, 2019) to identify the number of parent–offspring pairs (POPs) in the Lubbock River system. This analysis was conducted with the individuals collected within the ~1 km section of the Lubbock system that was enclosed between the two weirs, the section for which both adults and YOY were sampled. The following criteria were assumed as follows: female monogamy, male polygamy, and no inbreeding. COLONY was run five times. For each run, we summed the probabilities of each identified parent–offspring pair. We then used the median parent–offspring pair value across the five runs in the CKMR equation. We also compared the COLONY‐derived parent–offspring pair estimates with parent–offspring pair estimates obtained with CKMRsim (Anderson, 2020) using False Positive Rate (FPR) ~10 times smaller than the reciprocal of the number of comparisons.

## 3 RESULTS

### 3.1 Genetic quality control

The percentage of missing data per locus was *x* < 1% for 31 loci, 1% < *x* < 5% for 5 loci, and 5% < *x* < 10% for the remaining two loci, with an overall percentage of missing data per locus of 0.78%. Individuals exhibiting missing data at >7 loci were removed from analyses; this involved 155 individuals with the final data set considered in all subsequent analyses being *N* = 1,104 (507 adults and 460 YOY from the Lubbock system, and 137 YOY from the Blackstone system). There was no difference between H_o_ and H_e_ for any of the eight sampling locations (*α* ≥ 0.05) indicating there were no departures from Hardy–Weinberg Equilibrium (HWE). Sample size, however, varied among locations ranging between *N* = 49 and *N* = 594 (Table 1).

### 3.2 Population structure

**FIGURE 2:**
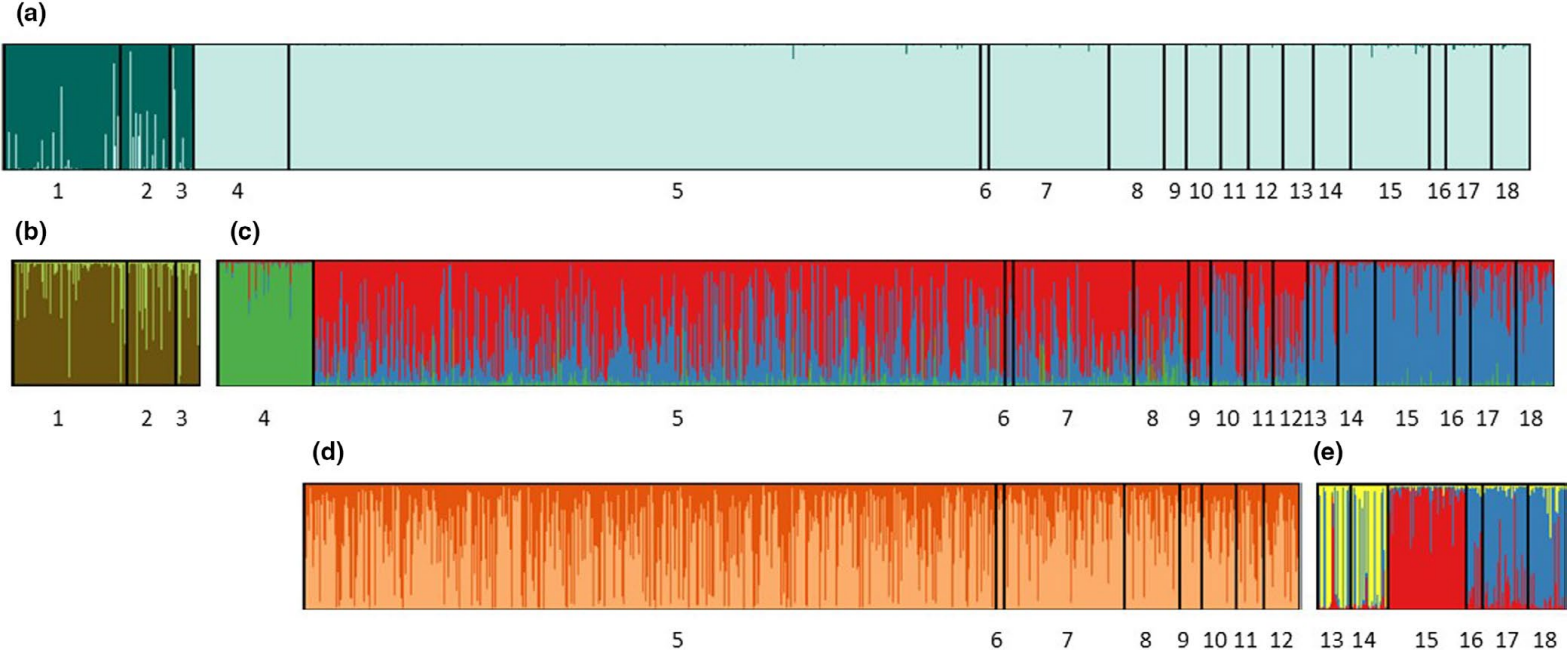
Hierarchical population structure (38 microsatellites) 1–3: Blackstone System, 4–18: Lubbock System, 4: Haunka Creek (Upstream of Weir), 5–7: Locations within Lubbock River Weirs, 8–18: Locations downstream of weir, 8–9: Snafu Creek, 10–12: Tarfu Creek, 13–14: Hitchcock Creek, 15: Shaker Creek, 16–18: Fourth of July Creek. (a) All samples combined, (b) Blackstone System, (c) Lubbock System, (d) Lubbock River and direct tributaries, (e) Tributaries of Atlin Lake

STRUCTURE first indicated *K* = 2 splitting the Blackstone and Lubbock systems (Figure 2a). Each group was then analyzed separately. We identified no further structure (i.e., *K* = 1) within the Blackstone system (Figure 2b). The collections from the Lubbock system were instead pooled into three groups (i.e., *K* = 3; Figure 2b). One group representing the fish upstream of the Lubbock River in a tributary to Little Atlin Lake (i.e., Haunka Creek) (Figure 2b). A second group encompassing all fish (adults and YOY) collected between the two weirs within the Lubbock River and two tributaries of the Lubbock River, both downstream of the weir (i.e., Snafu Creek, Tarfu Creek) (Figure 2b). A third group representing collections from three tributaries of Atlin Lake downstream of the Lubbock River (Figure 2b). Further analysis suggested all three downstream collections from tributaries of Atlin Lake (Hitchcock Creek, Shaker Creek, and Fourth of July Creek, samples 13–18 in Figure 2) were genetically distinguishable from each other (i.e., *K* = 3, Figure 2c) while no further structure was detected elsewhere. In total, therefore, six distinguishable populations were identified (Figure 2) with all individuals (adults and YOY) collected within the ~1 km weir‐enclosed section of the Lubbock River and the two downstream tributaries of Snafu and Tarfu creeks (i.e., locations 5–12 in Figure 2) grouping into a single population.

### 3.3 Effective population size and effective number of breeders

**FIGURE 3:**
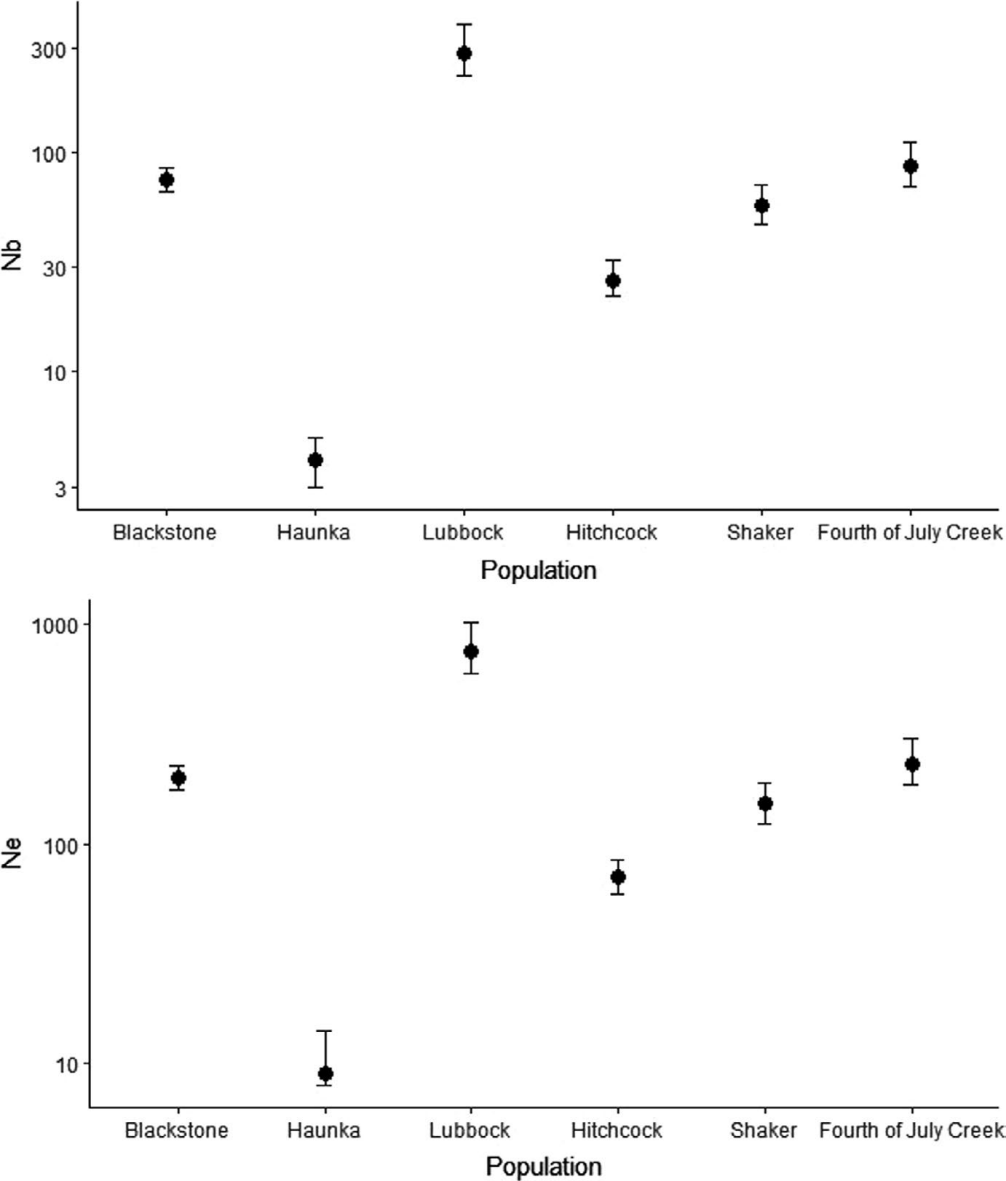
${\hat{N}}_{b(\mathrm{adj}2)}$ and ${\hat{N}}_{e(\mathrm{adj}2)}$ from raw *N_b_* estimates using LD based on 38 microsatellites: Blackstone—Blackstone River (3 sites), Haunka —Haunka Creek, Lubbock—Lubbock River YOY, Snafu Creek (2 sites), Tarfu Creek (3 sites), Hitchcock – Hitchcock Creek (2 sites), Shaker—Shaker Creek, Fourth of July Creek—Fourth of July Creek (3 sites). See numeric values in Table S2

The effective number of breeders (*N_b_*) and effective population size (*N_e_*) for the six identified populations are shown in Figure 3. The effective number of breeders and consequently the effective population size were considerably low for the Haunka Creek population (i.e., *N_b_* = 4 and *N_e_* = 9, Figure 3, see also Table S2).

### 3.4 Family sampling

The low effective number of breeders in the collection from Haunka Creek prompted us to examine family structure within that collection. We found that a small number of families each contributed disproportionate numbers of full sibs with the four largest families contributing 52 of 69 individuals representing ~75% of the individuals collected from Haunka Creek (Table 2).

**TABLE 2:** Haunka Creek number of full siblings per family as identified with COLONY using 38 microsatellite loci for Haunka Creek

Family	Number of full siblings
1	18
2	16
3	9
4	9
5	5
6	4
7	2
8	1
9	1
10	1
11	1
12	1
13	1

### 3.5 Census population size estimated by CKMR and $\hat{N}$_e_/$\hat{N}$_c_ ratio

**FIGURE 4:**
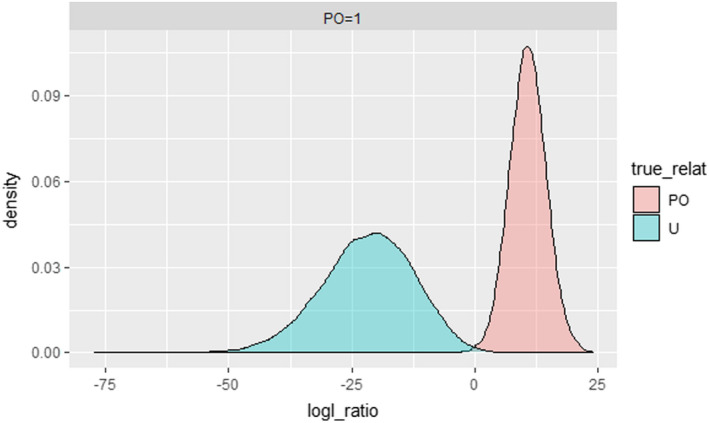
Distribution of the log‐likelihood ratio between parent–offspring pairs (PO) and Unrelated (U) individuals

Census population size was estimated for the population inhabiting the Lubbock River, and the two tributaries located downstream of the weirs (Snafu and Tarfu Creeks), all of which were identified as belonging to a single population in the STRUCTURE analysis (i.e., locations 5–12 in Figure 2). After filtering for number of missing loci and maturity, the final numbers of potential parents (mature adults) and juveniles considered in the parentage analysis were *N* = 413 and *N* = 87, respectively. The median number of parent–offspring pairs (POPs) found with COLONY is 37.67 (Table 3). Population abundance or *N_C_* for the Lubbock River was subsequently estimated as ${\hat{N}}_{C}\;=\;$1858 (Equation 3), with a CV = 0.1629 resulting in a 95% Confidence Interval CI = 1,259–2,457 (Table 4). The $\hat{N}$ *_e_*/$\hat{N}$ *_c_* ratio based on $\hat{N}$_e_ = 754 (592–1013) is $\hat{N}$ *_e_*/$\hat{N}$ *_c_* = 0.410. The number of POPs found with CKMRsim is 26 or 38.6 taking into consideration the False Negative Rate of 0.487, which translates into $\hat{N}$ *_c_* = 1812, CV = 0.1608 resulting in a 95% CI = 1,229–2,389. Figure 4 shows the distribution of log‐likelihood ratios for parent–offspring pairs versus that for unrelated individuals. The $\hat{N}$_e_/$\hat{N}$_c_ ratio then is = 0.421.

**TABLE 3:** Sum of probabilities for parent–offspring pairs (POP) output from COLONY based on 38 microsatellites for the Lubbock River

Run	Parent–Offspring Pairs (POP)
1	37.67
2	36.85
3	38.08
4	35.71
5	38.76
Median	37.67

**TABLE 4:** Close‐kin mark‐recapture (${\hat{N}}_{C(\mathrm{CKMR})}$) estimate of census population size for Arctic Grayling in the Lubbock River for the year 2018. Estimate is given with 95% CI (assuming each parent is equally likely to produce offspring), probability that a parent is present in the sample, *N*_(offspring)_ & *N*_(mature)_, Median POP, and CV: coefficient of variation assuming POPs follow approximately a Poisson distribution (Bravington et al., 2016)

	Lubbock River (COLONY)	Lubbock River (CKMRsim)
Probability parent in sample	0.15	0.15
*N*_(offspring)_ & *N*_(mature)_	87 & 413	87 & 413
Median POP	37.67	38.66
CV = 1/sqrt(median(POP))	0.1629	0.1608
${\hat{N}}_{C(\mathrm{CKMR})}$	1858	1812
(95% CI)	(1259–2457)	(1229–2389)

## 4 DISCUSSION

We have shown that Arctic Grayling (*Thymallus arcticus*) exhibit philopatry at the level of spawning aggregations as suggested by the population structure shown among grayling collections from different sampling sites in the Atlin Lake. Also, we show that tributaries in very close proximity to one another tend to display intermixing made evident by the absence of population structure between the collections from the weir‐enclosed section of Lubbock River and the two small tributaries (i.e., Snafu Creek and Tarfu Creek) downstream of the weirs in nearby locations. Further, we have used the close‐kin mark‐recapture approach to estimate the size of the population spawning in a single season within the ~1 km long enclosed section of the Lubbock River. These findings exemplify how genetics can be used to assist in the development of conservation and management initiatives for Arctic Grayling throughout the Yukon Territory. We describe our results in detail below.

We have shown that a large panel of sequenced microsatellite loci is effective for assessing population status and estimate population abundance. The cost and timeframe of developing microsatellite loci have decreased with improvements in sequencing technologies and availability of bioinformatic tools (Abdelkarim et al., 2009; Zhan et al., 2017). Ambiguity of scoring microsatellite loci has been eliminated through sequencing methods, which facilitate the precise scoring of base pair numbers (Darby et al., 2016). The scoring process can be automated using programs such as MEGASAT (Zhan et al., 2017) or Geneious software v. 9.1.8 (Kearse et al., 2012) with visual confirmation of scoring required.

Arctic Grayling are known to migrate from large rivers and lakes into smaller tributaries to congregate into spawning aggregations and, importantly, for the purpose of estimating population abundance, hatching takes place within two to three weeks after fertilization (Jessup & Millar, 2012). This allows for the assessment of parent–offspring relationships within a single spawning season provided two assumptions are met as follows: (a) adults are sampled randomly during the time the fish congregate for spawning and (b) the offspring, which are sampled as young‐of‐the‐year (YOY) are allowed adequate time to disperse throughout the system before collection to minimize the probability of a downward bias in the estimates of abundance that would result from the sampling of related individuals. In the present study, YOY were sampled with dipnets along the entire 1 km stretch of the Lubbock River enclosed by the two sets of weirs and in the two downstream nearby streams. This was done precisely to minimize the chances of such a downward bias stemming from the sampling of related individuals that had not yet dispersed through the system. Although CKMR is an effective method for the estimation of population abundance, it requires the fulfillment of a number of important assumptions including that individuals are thoroughly mixed and that they are sampled randomly (see Conn et al., 2020) with potential parents and offspring sampled independently of each other. When collections take place over several years, as we expect it to be the case in most scenarios, information on year of collection along with age and age‐specific fecundity and mortality rates is also required (See Bravington et al., 2016; Ruzzante et al., 2019).

The Lubbock River Arctic Grayling abundance estimate obtained with CKMR using COLONY (i.e., *N_c_*_(COLONY)_ = 1,858 (CV = 0.1629) was undistinguishable from that obtained with the CKMRsim parentage analysis R function once the false negative rate was taken into consideration (*N_c_*_(CKMRsim)_ = 1,812 (CV = 0.1608)). The close agreement between COLONY and CKMRsim in the estimates of the number of parent–offspring pairs is likely a consequence of the high reliability in the parentage assignment conferred by the large number of microsatellite markers used. All 26 of the parent–offspring pairs identified by CKMRsim were also detected by COLONY, while three other POPs detected by COLONY were also found by CKMRsim but exhibited probabilities that were marginally lower than the set false positive rate (FPR). Both estimates were, therefore, higher than the estimates obtained through snorkeler (*N_c_* = 91–505, Jessup & Millar, 2012) or angler surveys (*N_c_* = 600, Foos et al., 2014), and this is likely a result of our sampling protocol. We sampled all individuals entering or exiting the system over a 10 week‐long period. The snorkeler surveys instead, provide an instantaneous estimate (Jessup & Millar, 2012). CKMR estimates are independent of visibility and/or human disturbance that affects behavior. Therefore, CKMR is recommended for estimating *N_c_*, as a means of assessing population status. We do recognize, however, that CKMR abundance estimates for Arctic Grayling could be improved first by the collection of samples over a protracted period of time spanning several spawning seasons (years), an effort that would, in turn, require information on age (or size) specific fecundity and survivorship rates. For assessment of parentage using CKMRsim, we chose a log‐likelihood cut‐off of 10.7, which represents a false positive rate 10X lower than the reciprocal of the number of comparisons. This is the recommended false‐positive rate to avoid incorrectly identifying unrelated individuals as parent–offspring pairs (Anderson, 2020). All individuals identified in the 26 parent–offspring pairs were scored for all 38 loci. The number of parent–offspring pairs was then multiplied by the false negative rate of 0.487 to correct for potential‐related individuals that were not identified. We should note, however, that in this study we compared the distribution of parent–offspring relationships with only that of unrelated individuals and attempts to identify other potential relationships (e.g., uncle or aunts) were not pursued. If such relationships were found in our collections, they would be expected to exhibit relatively low log‐likelihood ratios probably below our cut‐off value of 10.7. They would thus be unlikely to be found among those included in the parent–offspring pairs. Table S3 shows one run of CKMRsim. In this particular run, there were 30 parent–offspring pairs identified, but 4 of them were dismissed because they involved two adult individuals.

The hierarchical population structure analysis provided evidence that the collections from Hitchcock Creek, Shaker Creek, and Fourth of July Creek, all small tributaries of Atlin Lake, are significantly distinguishable from each other. Thus, the hierarchical population structure analysis suggests philopatry for Arctic Grayling within Atlin Lake. Arctic Grayling migrate in late April through May from large rivers and lakes to small tributaries (Jessup & Millar, 2012). Our results are consistent with the hypothesis that mature individuals return to the same small tributary each spawning season. Temporal replicates would, however, be necessary to further test this hypothesis. Absence of temporal differentiation within tributary would confirm spawning philopatry.

Site fidelity or strong philopatry has been observed in closely related species including European Grayling, a species that, like Arctic Grayling, travels from large lakes or rivers to small tributaries to form spawning aggregations (Parkinson et al., 1999). Sockeye salmon exhibiting philopatry was shown to have greater fitness than immigrants from different systems (Peterson et al., 2014). Philopatric individuals tend to produce more offspring and have greater survival than immigrant individuals; however, immigrants are still of importance for maintaining intraspecific diversity (Peterson et al., 2014). Migrants maintain intraspecific genetic diversity of metapopulations through gene flow and admixture (Epps et al., 2005). Large increases in gene flow can prevent local adaptation due to the introduction of poorly adapted genes for the local environment (Hanski et al., 2011). Local adaptation could play an important role in differentiation of ecotypes due to phenotypic plasticity (Delgado et al., 2019).

We also estimated the effective number of breeders ($\hat{N}$ *_b_*) for each population. The effective number of breeders, *N_b,_* provides insight into eco‐evolutionary dynamics including mate choice and sexual selection which act on a single reproductive event (Waples & Antao, 2014). The effective number of breeders for Haunka Creek (population 2) was very low (i.e., $\hat{N}$ *_b_* = 4, from a sample size of 69). A low $\hat{N}$ *_b_* suggests a small number of parents contributed to the cohort sampled suggesting a departure from random mating (Hoarau et al., 2005). All individuals collected from Haunka Creek originate from a single location and the low $\hat{N}$_b_ estimate suggest family sampling is likely biasing the estimate downward. Indeed, we identified several full sib groups and found that 52 of 69 (~75%) individuals belonged to just four families. Family sampling is, therefore, the likely cause for the low $\hat{N}$ *_b_* estimate.

Effective population size*, N_e_*, was estimated to provide information for the assessment of population status. *N_e_* estimates provide the size of an idealized population exhibiting the same diversity as the one being studied across a generation or multiple cohorts (Palstra & Ruzzante, 2008). The effective population size, *N_e,_* was estimated for all populations using Equation (2) with estimates from ${\hat{N}}_{b(\mathrm{adj}2)}$ derived from raw ${\hat{N}}_{b(\mathrm{LD})}$. In general, all populations appeared to have sufficient genetic diversity, though Haunka Creek (${\hat{N}}_{e(\mathrm{adj}2)}$ = 9) was an exception. With this exception, all other *N_e_* estimates are higher than any of the thresholds proposed to avoid short‐term inbreeding depression (e.g., Frankham et al., 2014; Franklin, 1980).

Lastly, we estimated the $the\;{\hat{N}}_{e}/{\hat{N}}_{c}$ ratio for the Lubbock. This ratio reflects the proportion of the population that represents the genetic diversity within the population (Hedrick, 2005). The ratio of *N_e_/N_c_* can be used to evaluate factors that impact *N_e_* (see also Kovach et al., 2020; Waples & Feutry, 2021), where $\hat{N}$ *_e_* allows for assessment of extinction risk (Hedrick, 2005). Our estimate of *N_e_/N_c_* (0.41–0.42) is considerably higher than that obtained for a relict Arctic Grayling population in the southern edge of the species distribution (0.133; Kovach et al., 2020) and is also higher than the median estimate across taxa (0.14, Palstra & Ruzzante, 2008) indicating little concern for genetic diversity within the Lubbock River population.

Although the methods of this study were effective for assessing population status (${\hat{N}}_{e}$, ${\hat{N}}_{c}$ and their ratio ${\hat{N}}_{e}/{\hat{N}}_{c}$), there is a need for more accurate life‐history information and for temporally replicated sampling spanning several spawning seasons. Age‐specific fecundity and survival should be collected for the system of interest to improve accuracy of CKMR estimates. Age at maturity and adult lifespan has been shown to vary across a narrow geographic range in Alaska Arctic Grayling (Clark, 1992). Temporally replicated sampling is further needed for the assessment of the potential incidence of skip breeding and its influence on genetic and census estimates (Waples & Feutry, 2021) and of trends in population abundance. Thus, life‐history information and temporal replication are crucial for assessing population status accurately. In conclusion, we have shown how genetic methods can be used to improve our knowledge of population status, not just via the estimation of population structure but also through the estimation of effective and census population size. Such knowledge can be used to inform robust management and conservation policy of Arctic grayling in Yukon.

## CONFLICT OF INTEREST

Authors declare no conflict of interest.

## AUTHOR CONTRIBUTION

**Samuel Prystupa:** Data curation (equal); Formal analysis (equal); Writing‐original draft (equal). **Gregory Richard McCracken:** Data curation (equal); Methodology (equal); Writing‐review & editing (equal). **Robert Perry:** Conceptualization (equal); Investigation (equal); Resources (equal); Writing‐review & editing (equal). **Daniel Ruzzante:** Conceptualization (lead); Formal analysis (supporting); Funding acquisition (equal); Investigation (equal); Methodology (equal); Project administration (lead); Resources (equal); Supervision (lead); Writing‐review & editing (lead).

## Supporting information

Supplementary Material

## Data Availability

Microsatellite genotypes are available in the DRYAD Digital Repository https://doi.org/10.5061/dryad.2bvq83bpq.
