# Supplementary material for: Population abundance in arctic grayling using genetics and close‐kin mark‐recapture
Source: Ecol Evol. 2021 Apr 2;11(9):4763–73. doi: 10.1002/ece3.7378 (PMC8093667; doi:10.1002/ece3.7378)
Supplement: Supplementary file 1 — Supplementary Material [file ECE3-11-4763-s001.docx]

**APPENDIX**

***Molecular Protocol:***

A single DNA sample, chosen for quality and quantity of DNA, was used for the microsatellite loci development/primer design process. We followed the methods by McCracken et al. found in supplement MOESM3 in Yu et al., 2018. A single multiplex PCR was conducted with all 38 sets of primers that were kept for each individual sample using a Qiagen Multiplex PCR Kit (Qiagen Inc., Valencia, CA, USA) following the manufacturers guidelines scaled down to a total volume of 5µl. Multiplex PCRs were performed on Eppendorf (Hamburg, Germany) Mastercycler ep384 PCR machines using the following cycling conditions: 94⁰C for 15 minutes (m), 20 cycles o f 94⁰C for 30 seconds(s), 57⁰C for 180s, 72⁰C for 60s, and 68⁰C for 30m. Multiplex PCR product was diluted with 20µl double distilled water (ddH_2_O) to prepare samples for the subsequent index PCR.

Index PCRs were performed in 5µl reactions composed of 0.3µl of diluted multiplex PCR product, 0.2µM of two independent indices, 0.5µl of 10X buffer, 2.15µl of ddH_2_O, 0.2mM of each dNTPs, 0.5µl of MgSO_4_, and 0.25U of TSG polymerase (Bio Basic, Markham, ON, Canada). Index PCRs were performed on Eppendorf (Hamburg, Germany) Mastercycler ep384 PCR machines using the following criteria: 95⁰C for 120s, 20 cycles of 95⁰C for 20s, 60⁰C for 60s, 72⁰C for 60s, and 72⁰C for 10 minutes. Following the index PCRs individuals were pooled for cleaning and sequencing (see Zhan et al., 2017 for details).

The Index PCR product was cleaned following a bead cleaning protocol. Sera-Mag Speedbeads (see Faircloth & Glenn, 2011 for details) and pooled PCR product were combined with a ratio of 1.8:1 Sera-Mag Speedbeads (GE Healthcare, Little Chalfront, UK) to pooled PCR product. The mixture was then left to incubate for 15 minutes, followed by placement on a magnet for 5 minutes. The supernatant was then removed and discarded. Two 80% ethanol washes were carried out with the following protocol: add 200µl of 80% ethanol, place on magnet for 30 seconds, remove supernatant and ensure no solution remains in the tube. Next, 52.5µl of Tris Tween was added, the solution was briefly vortexed and allowed to incubate for 2 minutes, allowing time for the DNA to elute from the beads to resuspend beads and allow the previously bound DNA to elute. The solution was the once again placed on a magnet for 5 minutes. The supernatant, containing only clean pooled library (DNA), was removed and stored in a tube to prepare for sequencing.

The clean pooled library was then quantified on a Roche LC480 qPCR machine (Roche, Basel, Switzerland) using a Kapa Library Quantification Kit (Roche, Pleasanton, California) following the manufactures instructions. Given the quantification value, the pooled library was denatured and diluted appropriately to 15pM for use on an Illumina MiSeq Benchtop Sequencer (Illumina, San Diego, CA, USA) using MiSeq 150 cycle V3 chemistry kits and selecting for dual indexing due to the nature of our library preparation.

**References:**

Yu D, Ding X, Zhang Z, Zeng Y, Liu H, Wang L, …, Zhang Q. 2018. Correction to: Microsatellite records for volume 10, issue 2. *Conservation Genetics Resources.* 1-2. <https://doi.org/10.1007/s12686-018-1056-6>

**Table S1**. Locus name, primer sequences, motif, number of alleles, average number of alleles per population, and allele size range for the 38 microsatellite loci that were kept.

| **Locus Name** | **Left Primer Sequence** | **Right Primer Sequence** | **Motif** | **Number of Alleles (Entire Dataset)** | **Average Number of Alleles Per Population** | **Allele Size Range (Entire Dataset)** |
| --- | --- | --- | --- | --- | --- | --- |
| TAR2 | GAAAGGAAGATGGCACCCTC | AGCGAGGAGGAAAGGGATTG | CT | 6 | 3.667 | 21-35 |
| TAR3 | GCTCTGACCAGTACCTCTCC | TGTGGGAAGTCTATGTGGCC | AC | 7 | 4.167 | 21-33 |
| TAR9 | TCTGGGACTAGAGTGTGTGG | CTTCTCTGGGCTGTCATGTTG | GT | 23 | 10.167 | 22-84 |
| TAR12 | TCTGAACGGTTGGTGTGGTG | CAGGTCCGCTTTCTGGAATAG | GT | 4 | 2.333 | 28-48 |
| TAR13 | CGCAGGATTCCGACAATGAG | TACCTCCGCTGGATCAAAGG | GT | 8 | 4.000 | 28-48 |
| TAR14 | AGCTCAAGATCAATAGGGAAGG | TCTCCTGTCAACCCTACACTC | GT | 13 | 5.333 | 21-49 |
| TAR15 | GCCCTTTCAATACCACGCTC | ATGTCGCATCATCCACCCAC | CT | 3 | 2.167 | 29-33 |
| TAR18 | ACTGACCGTGTGTTATTTCCC | GACAGCCCAGCCTGATATAC | AC | 4 | 2.833 | 27-37 |
| TAR22 | CATCCACCGCTTCGTTTCC | CACACAGACCAAGAAATCATGC | CT | 5 | 2.833 | 33-41 |
| TAR26 | AGCGACAACACTGAACTATCAC | CATGGTCAATCTGGTAAGCCC | GT | 5 | 2.833 | 25-33 |
| TAR28 | CGTGTGTATGTCTTTCGCTCC | GCAGTATATGTAGCCCAACTCG | AC | 24 | 10.833 | 23-75 |
| TAR29 | TGCAGTTGACAGTGAGGATG | ACACACGCCAAACTCAGGAG | GT | 8 | 4.500 | 32-48 |
| TAR30 | TGCGACACGTAACTCCTCTC | ACACCCACAGCCTTTCAATC | CT | 8 | 3.000 | 22-48 |
| TAR31 | TGGCTCTTCACTAGGTCCTG | ACTAACGCACACCTCTCTCC | AG | 15 | 6.333 | 34-70 |
| TAR34 | GCAGCAGAGGGAAACAGAAAC | TTCACTCCCTCCACCACTTC | AG | 8 | 5.000 | 32-52 |
| TAR39 | CAATACCTCATGCCTCGCTC | TGATCATCACCGAGAGACCG | CT | 7 | 2.833 | 29-49 |
| TAR40 | TGAGGTCTAACTACAAAGCACC | TGTATGCCAACCTTGAGTCTC | GT | 4 | 2.333 | 36-44 |
| TAR41 | GGTTATCAAGATCCAGGCGTC | TGCCTGCTGTTATTACGTTGC | AC | 9 | 5.167 | 26-42 |
| TAR44 | CTCCTGTTCTACATTGAGGTGC | GAGAGTGGCAGGGATTAGGG | CT | 7 | 3.500 | 35-47 |
| TAR47 | GCCTGTGTGTTTCCTGACTG | CCACTGGATGCTCTTTATGGTG | AC | 6 | 3.333 | 40-52 |
| TAR50 | GAAAGATCTGATTGCGGGCG | ACATGCAGACTCCACACTCC | GT | 6 | 4.333 | 35-49 |
| TAR52 | CACTGCTCGAAGGTTACTCC | AGTGGGCAGAAGACAGAAGG | GT | 2 | 2.000 | 39-43 |
| TAR53 | GTGATTTGAGCAATGAGACGTG | ACAGGCACACACTAAGAAGC | GT | 5 | 2.500 | 35-43 |
| TAR54 | AGACTGCTGTGTTCCTCTACTC | GACCCACATTCACTTTCACCC | GT | 5 | 3.500 | 33-43 |
| TAR60 | AGACACCAACCACTTCAAAGG | AGAGGAACGGAGAGTGTAACAG | AC | 14 | 7.333 | 32-66 |
| TAR63 | AATCAATGCTGTGAGTGAAACC | CAATCTCTTTCCTGCCCGAG | CT | 10 | 5.167 | 43-65 |
| TAR64 | TCTGCTGTGAAACTCTGTATGG | TGCCGTGTGACTGTGCAG | AG | 5 | 3.167 | 41-51 |
| TAR65 | ACGCTCCTATGCTTCCTATTC | GGATACCATGACTAAGGGCTG | CT | 7 | 4.667 | 35-47 |
| TAR67 | GATCCTGGAACACTTGGCTG | GTATGACGCTCTGCTGTGAC | AC | 6 | 2.667 | 51-63 |
| TAR71 | GGCTGGGTGGTCTCTTTCTG | GCTGATAATGCCCACCTCTG | AC | 5 | 2.500 | 49-57 |
| TAR72 | TCACGTAAACACATGTCCACAG | TGAGGAGCGTCATCTGTGTG | AC | 4 | 2.167 | 51-57 |
| TAR86 | TAGGCGCATACACCACCTAC | CGATCCGCCAAGAAGCAC | CTT | 10 | 6.667 | 21-45 |
| TAR87 | CAAACAGCATGTCCCGGATG | ACGCCTGTACAACTCATCAC | ACT | 5 | 2.500 | 48-60 |
| TAR90 | CTGGCCCAGTTCCTCATCC | CATTTGGGATGTGGCAATAACC | ATT | 7 | 3.667 | 35-62 |
| TAR92 | GACTCTGAAAGGGATGCTGC | CCTAGGTGGGTGTTCTGAATG | GAT | 4 | 2.500 | 39-54 |
| TAR93 | GAGGACTCACTGCCAGGG | CCTACAGGTGGAGAACTACATC | GAT | 8 | 4.500 | 49-76 |
| TAR95 | CGCTTTAATGGGAAACACGC | GTGGAGTTGCAATGATGGGC | CTT | 10 | 5.500 | 53-83 |
| TAR96 | TCAGTCATTTGTGTCAGTGCC | AAATCCCTGCAATCGTCGTC | ACT | 9 | 4.500 | 37-70 |

**Table S2**. Estimates (95% CI) of the effective number of breeders ($\hat{N}$_b(LD)_) obtained with YOY individuals. Estimates of $\hat{N}_{b(adj2)}$ and $\hat{N}_{e(adj2)}$ are given assuming AL= 4.3 and $\propto$= 6 for the year 2018. n= sample size.

| Population | n | $\hat{N}$_b(LD)_ (95% CI) | $\hat{N}_{b(adj2)}$ (95% CI) | $\hat{N}_{e(adj2)}$ (95%CI) |
| --- | --- | --- | --- | --- |
| Blackstone River | 137 | 85 (75-97) | 75 (66-85) | 199 (176-227) |
| Haunka Creek | 69 | 4 (3-6) | 4 (3-5) | 9 (8-14) |
| Lubbock River | 213 | 322 (253-433) | 283 (222-380) | 754 (592-1013) |
| Hitchcock Creek | 49 | 30 (25-36) | 26 (22-32) | 70 (59-84) |
| Shaker Creek | 57 | 65 (53-81) | 57 (47-71) | 152 (124-190) |
| FOJC | 72 | 99 (80-128) | 87 (70-112) | 232 (187-300) |

**Number of Parent-Offspring pairs identified with CKMRsim**. 26 of the 30 Parent-Offspring Pairs (POPs) identified by CKMRsim with a loglikelihood ratio greater than 10.7 were kept (10X lower than the reciprocal of the number of comparisons). All POPs that identified two adults making a POP were removed from subsequent analysis because it is unlikely that an adult in the spawning population reproduced and survived until the offspring reached maturity. The 26 POPs identified were multiplied by the False Negative Rate of 48.7%. Thus, the number of POPs used for the CKMR equation is 38.66.

**Table S3.** Identified parent, identified offspring, loglikelihood ratio of the Parent-Offspring Pair, and the number of loci used to identify the pair using one of the CKMRsim runs. Note: in this particular run 30 Parent-Offspring pairs were identified, 4 of which were not considered because they involved two adult individuals.

| Pair | Parent ID | Offspring ID | logl_ratio | num_loci |
| --- | --- | --- | --- | --- |
| 1 | 6631-07-19 | L-047-8-22 | 19.94134 | 38 |
| 2 | 6753-27-19 | L-003-22-19 | 18.28797 | 38 |
| 3 | 6629-25-21 | L-018-21-20 | 16.36953 | 38 |
| 4 | 6628-28-34 | L-110-17-20 | 16.27672 | 38 |
| 5 | 6636-02-17 | L-006-25-19 | 15.01547 | 38 |
| 6 | 6592-18-19 | L-038-25-21 | 14.58177 | 38 |
| 7 | 6698-23-19 | L-021-24-20 | 14.38741 | 38 |
| 8 | 6811-32-17 | L-025-2-21 | 13.54007 | 38 |
| 9 | 6617-04-21 | L-042-3-22 | 13.52001 | 38 |
| 10 | 7009-33-18 | L-083-30-18 | 13.44477 | 38 |
| 11 | 6945-17-21 | L-046-7-22 | 13.37208 | 38 |
| 12 | 7011-24-27 | L-082-29-18 | 13.28508 | 38 |
| 13 | 6765-04-17 | L-016-19-20 | 13.08833 | 38 |
| 14 | 6621-32-17 | L-029-6-21 | 13.07083 | 38 |
| 15 | 6589-22-34 | L-010-3-20 | 12.80309 | 38 |
| 16 | 6470-05-22 | L-031-8-21 | 12.01473 | 38 |
| 17 | 6896-08-21 | L-005-24-19 | 11.98722 | 38 |
| 18 | 6489-14-17 | L-029-6-21 | 11.86299 | 38 |
| 19 | 7003-14-20 | L-016-19-20 | 11.70974 | 38 |
| 20 | 6463-07-18 | L-092-15-19 | 11.61683 | 38 |
| 21 | 6956-07-22 | L-102-33-19 | 11.49839 | 38 |
| 22 | 6592-18-19 | L-019-22-20 | 11.20894 | 38 |
| 23 | 6470-05-22 | L-020-23-20 | 10.97069 | 38 |
| 24 | 6865-32-18 | L-081-28-18 | 10.89337 | 38 |
| 25 | 6482-21-18 | L-057-11-17 | 10.85814 | 38 |
| 26 | 6598-18-20 | L-050-21-22 | 10.70183 | 38 |
|  | Adult ID | Adult ID | Log_l ratio | Num_loci |
| 27 | 6474-31-33 | 6493-31-33 | 14.67674 | 38 |
| 28 | 6508-34-33 | 6535-34-33 | 12.26922 | 38 |
| 29 | 7445-08-21 | 7487-31-20 | 11.99699 | 38 |
| 30 | 6941-28-20 | 7497-10-20 | 11.05324 | 38 |
